# In-Depth Proteomic Analysis of the Hippocampus in a Rat Model after Cerebral Ischaemic Injury and Repair by Danhong Injection (DHI)

**DOI:** 10.3390/ijms18071355

**Published:** 2017-06-24

**Authors:** Yiran Cui, Xin Liu, Xianyu Li, Hongjun Yang

**Affiliations:** 1 Institute of Chinese Materia Medica, China Academy of Chinese Medical Sciences, 100700 Beijing, China; cui_1ran@163.com (Y.C.); xinliu1011@126.com (X.L.); 2 Beijing Key Laboratory of Traditional Chinese Medicine Basic Research on Prevention and Treatment for Major Diseases, Experimental Research Center, China Academy of Chinese Medical Sciences, 100700 Beijing, China

**Keywords:** hippocampal region, cerebral ischaemic injury, proteomic analysis, Danhong injection (DHI)

## Abstract

Stroke is the second most common cause of death worldwide. A systematic description and characterization of the strokes and the effects induced in the hippocampus have not been performed so far. Here, we analysed the protein expression in the hippocampus 24 h after cerebral ischaemic injury and repair. Drug intervention using Danhong injection (DHI), which has been reported to have good therapeutic effects in a clinical setting, was selected for our study of cerebral ischaemia repair in rat models. A larger proteome dataset and total 4091 unique proteins were confidently identified in three biological replicates by combining tissue extraction for rat hippocampus and LC-MS/MS analysis. A label-free approach was then used to quantify the differences among the four experimental groups (Naive, Sham, middle cerebral artery occlusion (MCAO) and MCAO + DHI groups) and showed that about 2500 proteins on average were quantified in each of the experiment group. Bioinformatics analysis revealed that in total 280 unique proteins identified above were differentially expressed (*P* < 0.05). By combining the subcellular localization, hierarchical clustering and pathway information with the results from injury and repair phase, 12 significant expressed proteins were chosen and verified with respect to their potential as candidates for cerebral ischaemic injury by Western blot. The primary three signalling pathways of the candidates related may be involved in molecular mechanisms related to cerebral ischaemic injury. In addition, a glycogen synthase kinase-3β (Gsk-3β) inhibitor of the candidates with the best corresponding expression trends between western blotting (WB) and label-free quantitative results were chosen for further validation. The results of Western blot analysis of protein expression and 2,3,5- chloride three phenyl tetrazole (TTC) staining of rat brains showed that DHI treatment and Gsk-3β inhibitor are both able to confer protection against ischaemic injury in rat MCAO model. The observations of the present study provide a novel understanding regarding the regulatory mechanism of cerebral ischaemic injury.

## 1. Introduction

Stroke is the second most common cause of death worldwide: approximately 6.5 million stroke patients die annually [1]. Cerebral ischaemic stroke, the major type of stroke, accounts for approximately 80% of stroke deaths. However, there are also 25.7 million stroke survivors [2,3]. Cerebral ischaemia or stroke can lead to broad cerebral injury and result in high disability and mortality rates in many countries [4]. Cognitive deficit is widely found in stroke survivors and progresses into dementia in later phases [5,6]. The loss of memory is one important cause of dementia after stroke. Therefore, systematic investigations of the complex pathological cascades during ischaemic brain injury can help develop effective treatment and elucidate novel therapeutic targets against cerebral ischaemia [2,3]. Tremendous progress has been made in the understanding of the fundamental mechanisms of neuronal cell death. It is known that pathological events during ischaemic stroke include inflammation, excitotoxicity, mitochondrial depolarization, oxidative stress, and apoptosis [7]. However, the translation of these powerful molecular and cellular principles into clinically effective neuroprotective therapies in stroke has been challenging.

The hippocampus is the major region responsible for memory formation and consolidation [8], and is also the most sensitive region for cerebral ischaemic injury [9]. Cerebral ischaemic strokes occur when blood flow to the brain stops or is reduced during cardiac arrest or respiratory arrest, and the ischaemic insults lead to irreversible neuronal damage in some regions, including the cerebral cortex, hippocampus and striatum [10,11]. The CA1 region of the hippocampus is known to be highly impressionable to transient global cerebral ischaemia [12], and neuronal loss in the hippocampal CA1 region gradually occurs three to four days after transient ischaemic injury, a phenomenon that has commonly been termed “delayed neuronal death” [9]. Possible mechanisms related to the delayed neuronal death following transient global cerebral ischaemia are proposed to involve excitotoxicity, oxidative stress and inflammation [13,14,15]. However, the precise intrinsic mechanisms leading to delayed neuronal death are not yet clearly understood.

Danhong injection (DHI), a Chinese Materia Medica standardized product extracted from *Radix Salviae miltiorrhizae* and *Flos Carthami tinctorii*, has been shown to be effective in protecting against ischaemic stroke [16,17,18,19]. However, its pharmacological mechanisms are still unclear [20].

Mass spectrometry (MS)-based proteomics is a powerful tool that provides insights into the spatiotemporal patterns of protein expression [21]. Label-free quantitative approaches have been performed in many studies and are promising alternatives to stable isotope labelling. They are fast, easy to perform, and inexpensive, and they allow a higher dynamic range. Furthermore, any soluble biological material can be used, and unlimited numbers of samples can be compared [22,23,24].

Here, we first established a stable and reliable rat model of cerebral ischaemic injury, and evaluated the pharmacological effects of DHI on middle cerebral artery occlusion (MCAO) rat. Then, we reported a comprehensive delineation of the proteome in four experimental groups (Naive, Sham, MCAO, MCAO + DHI) that differ with respect to cerebral ischaemic injury and repair by combining tissue extraction for rat hippocampus and liquid chromatography-mass spectrometry (LC-MS/MS) for protein identification. A label-free approach was then used to quantify the differences in the signal intensity of the MS results among the four experiment groups. Hierarchical clustering and bioinformatics analysis revealed the differentially expressed proteins, which potentially involved in the development of cerebral ischaemic injury phase and repair phase. The present study provides the first evidence that some important signalling pathways related to the cerebral ischaemic injury in vivo and the stimulus-related proteins of DHI treatment supported the long-term potentiation in the rat hippocampus after cerebral ischaemic injury.

## 2. Results

### 2.1. Experimental Workflow

**Figure 1:**
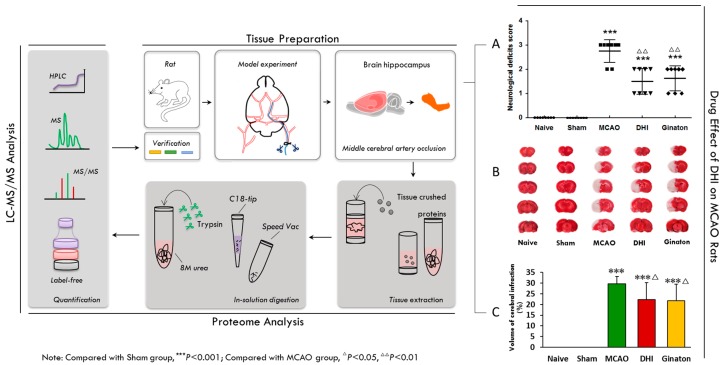
Overview of the experimental workflow. Firstly, permanent occlusion of the middle cerebral artery (MCAO) was established in rats with an intraluminal silicon-coated filament (tissue preparation). Secondly, to ensure the reliability and rationality of the rat model of cerebral ischemic injury, an initial experiment revealed the success of the establishment of focal ischemic models (A,B,C). Thirdly, the hippocampal region of the model brains was tissue crushed and the protein extracted, after injury time of 24 h in focal ischemic models (tissue preparation). Fourthly, proteins for proteome analysis were tryptic digested in solution, desalted using a C18 pre-column, and subjected to LC and Q-Exactive analysis (proteome analysis). Finally, the peptide mixture was analyzed with online reverse-phase chromatography and mass spectrometry and a label-free approach was used for the quantitative analysis (LC-MS/MS Analysis). (**A**) The results of neurological deficits Score shown that DHI and Ginaton can improve neurological function in MCAO rats after 24 h ischaemic injury (Table 1, the date was tested by a rank sum test); (**B**,**C**) 2% TTC staining results and histogram shown that DHI and Ginaton can decrease the volume of cerebral infraction in MCAO rats. The results of TTC were tested by one way ANOVA and multiple testing (Table 2).

Figure 1 is a schematic representation of our experimental approach. To investigate the proteins that are related to cerebral ischaemic injury, we followed this workflow to profile the differential protein expression in the rat hippocampal region after permanent cerebral ischaemic injury. In brief, to ensure the reliability and rationality of the rat model of cerebral ischaemic injury, permanent occlusion of the middle cerebral artery (MCAO) was established in rats with an intraluminal silicon-coated filament [25,26,27]. An initial experiment demonstrated the successful establishment of focal ischaemic models [28,29,30]. Thus, an injury time of 24 h in a focal ischaemic model was identified as the essential conditions for our experiment [27]. The hippocampal region of the rat brain tissue was homogenized, and the protein was extracted. For the proteome analysis, proteins were tryptic-digested in solution, desalted using a C18 pre-column, and subjected to LC and Q-Exactive analysis. To ensure the reliability of the quantitative profiling results, the samples were prepared and fractions were collected on three independent occasions (three biological replicates). The MS data were searched against the UniProt rat database (20150320). The number of peptide spectral matches (PSMs) and the matched precursor ion area were further independently used for label-free quantification. Here, we have used DHI treatment to facilitate the repair of cerebral injury in rats after the MCAO operation, as numerous clinical observations have indicated favourable outcomes for stroke patients receiving DHI therapy [16,17,18,19]. DHI, obtained from Shandong Danhong Pharmaceutical Co., Ltd (Zibo, China), is a Chinese Materia Medica standardized product extracted from *Radix Salviae Miltiorrhizae* and *Flos Carthami tinctorii* with a raw material dose ratio of 3:1 [31].

### 2.2. Evaluation of the Pharmacological Effects of DHI on MCAO Rats

To experimentally validate the protein response to the DHI-mediated protection against cerebral ischaemia, first, the pharmacological effects of DHI on MCAO rats were evaluated. As shown in Figure 1A, 24 h after the MCAO operation, Longa’s Neurological Severity Score of the model group revealed remarkable ischaemic injury (*P* < 0.001), while the groups that received either DHI or the positive control, ginaton, scored observably lower (*P* < 0.01). The scores of neurological deficit were analyzed using a rank sum test [32], which indicated improved neurological function in MCAO rats (Table 1). Moreover, rat brains were stained with 2% TTC. The ischaemia produced a marked infarct as a result of the MCAO operation in the serial coronal brain sections. The mean infarct volume in the model group was 29.71 ± 3.47 (%) (*P* < 0.001) (Table 2). Intraperitoneal injection of ginaton and DHI (0.45, 0.72 mL/kg) significantly reduced the infarct volume (*P <* 0.05) compared to the model group. As shown in Figure 1B,C, 2% TTC staining of the brains from the rats treated with DHI showed a lower degree of ischaemic injury.

### 2.3. Proteome Profiling of the Hippocampus in Four Groups of Rats

By combining special tissue extraction for rat hippocampus and LC-MS/MS for protein identification, a large proteome dataset with 4091 unique proteins was confidently identified in three biological replicates. A label-free approach was then used to quantify the differences in the signal intensity of the MS results among the four experiment groups (Table S1, sheet 1: Quantification). Totally 280 significant unique proteins were obtained by statistics tests of these experiment groups (Naive vs. Sham, Sham vs. MCAO and MCAO vs. MCAO + DHI).

**Figure 2:**
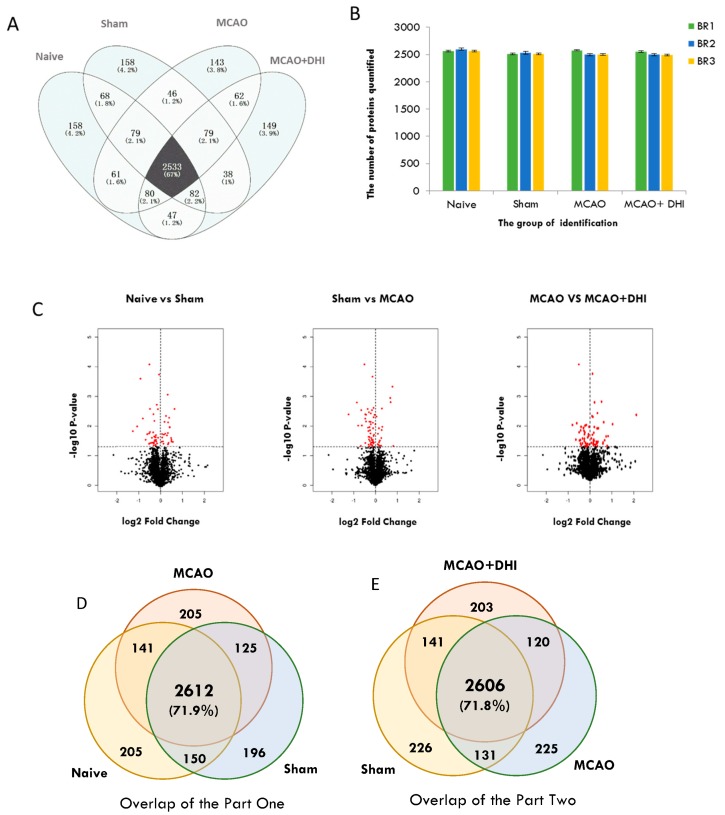
Summary of the identification and quantitative analysis of the hippocampal proteins. (**A**) Overlap of highly confident hippocampal proteins (4091 unique proteins) identified between the different rat groups (Naive, Sham, MCAO, MCAO + DHI); ~70% of these proteins were in common; (**B**) the number of label-free quantified proteins in each hippocampus group with three replications (BP: Biological repeat) and ~2500 proteins quantified on average for each run; (**C**) proteins with significantly altered expression were selected by *t*-test using SPSS and shown in red plots of the volcano from these groups (Naive vs. Sham: 123, Sham vs. MCAO: 122, MCAO vs. MCAO + DHI: 85); (**D**,**E**) Overlap of highly confident proteins identified from part one and part two (we divided the experimental groups into two parts for the next analysis; part one contained the Naive, Sham and MCAO groups and part two contained the Sham, MCAO and MCAO + DHI groups).

Combining the raw data from 12 MS runs (four groups, three biological replicates), 4091 proteins (Mascot ion score > 20, peptide false discovery rate (FDR) < 1%) were identified in the rat hippocampal proteome (Figure 2) (Table S1, sheet 2: Identification). Of these proteins, 2533 (~70%) were common among the four groups (Naive, Sham, MCAO, MCAO + DHI) (Figure 2A) and each of the sets shown in the figure represents proteins identified in at least one of the three biological replicates. The differentiable and equivalent proteins with more than one unique peptide were retained. In each group, we identified and quantified ~2500 proteins on average (Figure 2B). Next, we tested significant protein expression between the four groups with three biological replicates using SPSS analysis (*t-*test of Naive vs. Sham, Sham vs. MCAO, MCAO vs. MCAO + DHI, *P* < 0.05 was considered significant), and the expression of 280 proteins (Naive vs. Sham: 123, Sham vs. MCAO: 122, MCAO vs. MCAO + DHI: 85) exhibited significant differences (Figure 2C) (Table S1, sheet 3: Mean + SD).

### 2.4. Annotation Map and Profiling of the Proteome

**Figure 3:**
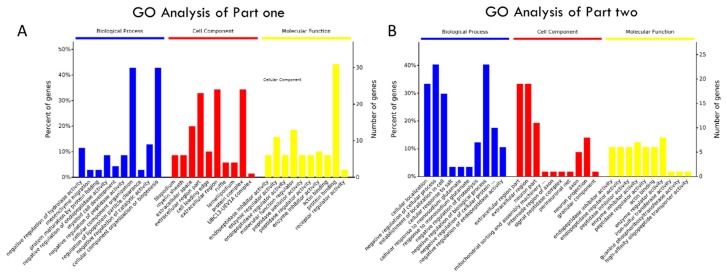

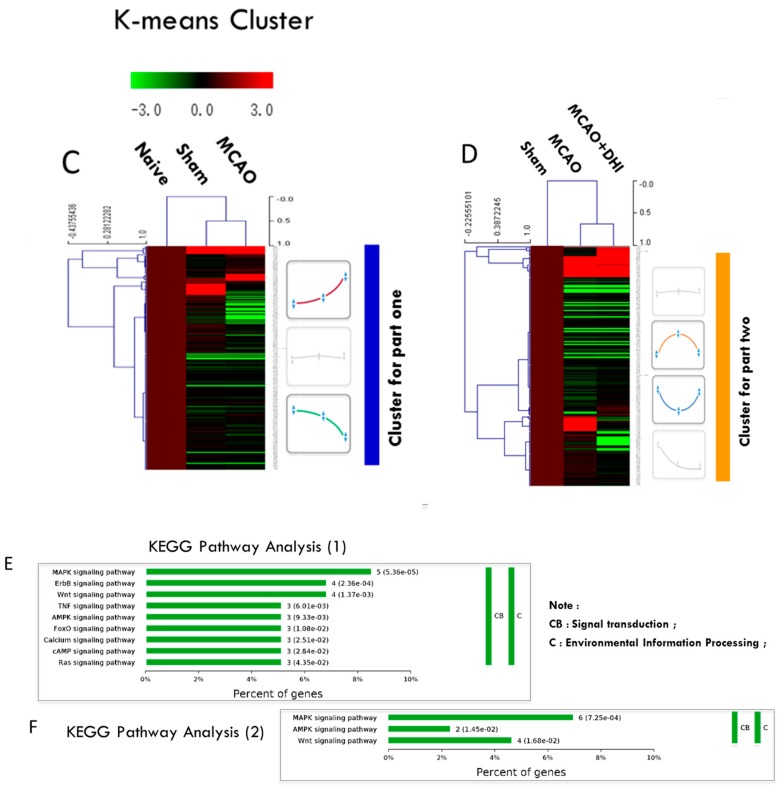
Gene Ontology (GO)/KEGG pathway analysis and K-means clustering analysis of hippocampus proteins with significantly altered expression. (**A**) GO annotation and enrichment of the hippocampus proteins with significantly altered expression in part one; (**B**) GO annotation and enrichment of the hippocampus proteins with significantly altered expression in part two; (**C**) clustering of significant altered hippocampus proteins of part one, and the two major clusters extracted by K-means clustering for the next analysis; (**D**) clustering of significantly altered hippocampus proteins of part two, and the two major clusters extracted by K-means clustering for the next analysis; (**E**) the enriched biological pathways in these two major clusters of significantly altered proteins in part one, as viewed by KEGG pathway analysis; (**F**) KEGG pathway analysis of only enriched pathways related to signal transduction and environment for these significantly altered proteins of part two.

We wished to investigate whether our proposed proteome includes key factors or mediators that may be related to injury and repair in the hippocampus during the development and progression of cerebral ischaemia. Therefore, we divided the proteome subset into two parts. The first part focused on injury, and contained the Naive, Sham and MCAO groups, while the second part focused on repair, and contained the Sham, MCAO and MCAO + DHI groups. We then performed an enrichment map profile analysis of our rat hippocampus proteome quantification dataset. Gene Ontology (GO)/Pathway enrichment analysis of the two proteome subsets revealed high consistency between proteome features and the physiological activities of each group. For the injury group of proteins, GO annotations for biological processes mapped 225 significant proteins from 2612 common overlapping proteins (Figure 2D) (Table S1, sheet 4: GO for part one). From the classification chart analysis [33], these proteins were separated based on location into the categories of extracellular regions, the extracellular region part and the extracellular space (32%, 30% and 20%, respectively), while protein complex (nearly 40%) and other categories were also represented in the hippocampal proteome dataset through the enrichment analysis of the Naive, Sham and MCAO groups (Figure 3A). For part two, the repair group, to obtain a general overview of the cellular localizations and molecular functions of the identified proteins, we analysed the 184 significantly differentially expressed proteins of the 2606 common overlapping proteins using GO and KEGG pathway enrichment (Figure 2E). From the GO analysis, we observed that the top enriched categories of cellular localization were related to extracellular regions (32%) and extracellular region part (32%), which could include proteins involved in cell processing due to changes in environmental information (Figure 3B) (Table S1, sheet 5: GO for part two). The original data of GO annotations above were obtained from OmicsBean analysis tools. Meanwhile, we re-calculated the GO annotations data using DAIVD software and, in brief, 36.9% of proteins in part one (*P*-value = 1.2 × 10^−20^, Benjamini FDR = 4.2 × 10^−18^) and 40% of proteins from part two (*P*-value = 1.5 × 10^−24^, Benjamini FDR = 5.4 × 10^−22^) were localized as the extracellular exosome. Among the most common cellular localizations of the repair proteins, the top two enriched categories were “extracellular region”, “extracellular space” or “extracellular part”, which further indicated that the dataset is a high-quality collection of secretory proteins. Secretory proteins are a crucial part of the extracellular matrix that creates an environment that is favourable to the disorder in many diseases [34].

### 2.5. Hierarchical Clustering of the Significantly Altered Proteins

A significant difference (*P* < 0.05) of the 280 total proteins in at least one of the four groups (Naive, Sham, MCAO, MCAO + DHI) was taken as an excellent basis for further statistical analysis. We then performed hierarchical clustering based on Pearson’s correlation of quantitation and the K-means values of the proteins per group.

For part one (225 proteins), three main clusters can be observed, with upregulated, downregulated or unchanged proteins (Table S1, Sheet 6: Cluster for part one). In cluster 1, which includes 114 proteins, the expression level of the proteins continuously increased with the progression through the Naive, Sham and MCAO groups. Cluster 2, which contains nearly one-tenth of the identified altered proteins, is a group with mixed regulation and without any significant changes in abundance among the three groups. Cluster 3, which represents nearly 41% of the dataset, exhibits a trend of expression that was continuously downregulated in the MCAO group and includes 91 proteins, similar to cluster 1 (Figure 3C).

For part two, we used the same method of hierarchical and K-means clustering to filter the 184 significant commonly expressed proteins (Table S1, Sheet 7: Cluster for part two). Four clusters can be observed and only two important clusters were used for our next analysis, one in which the protein expression level first increased and then decreased and another in which the expression level was first decreased and then increased. In the former cluster, the expression of 16 proteins increased in the MCAO group compared to the Sham group and then decreased in the MCAO + DHI experimental group. In the latter cluster, the protein expression was higher in the Sham and MCAO + DHI groups and downregulated in the MCAO group. This second cluster includes less than one-tenth (17) of the significantly altered proteins. (Figure 3D).

To investigate this more systematically, we analysed these hierarchical clusters: cluster 1 (114 proteins) and cluster 3 (91 proteins) of part one, clusters 2 (16 proteins) and cluster 3 (17 proteins) of part two. Next, we used these proteins as an input to view enrichment pathways by KEGG analysis.

### 2.6. Pathway Enrichment of the Significantly Altered Proteins

We examined which molecular pathways these proteins (chose part one: 205 proteins, part two: 33 proteins) may be involved in using KEGG pathway analysis by OmicsBean analysis tools. In particular, we primarily chose pathways related to signal transduction and environmental information processing, which may be more closely related to the actual in vivo changes in the hippocampus after cerebral ischaemic injury. For the injury subset of proteins, the pathways “MAPK signalling” (*P*-value = 5.36 × 10^−5^), “ErbB signalling” (*P*-value = 2.36 × 10^−4^), “WNT signalling” (*P*-value = 1.37 × 10^−3^), “TNF signalling” (*P*-value = 6.01 × 10^−3^), “AMPK signalling” (*P*-value = 9.33 × 10^−3^), “FoXO signalling” (*P*-value = 1.08 × 10^−2^), “Calclum signalling” (*P*-value = 2.51 × 10^−2^), “cAMP signalling” (*P*-value = 2.84 × 10^−2^) and “Ras signalling” (*P*-value = 4.35 × 10^−2^) were enriched, based on the KEGG pathway analysis results (Figure 3E). The first five pathways enriched were the most significant (*P*-value < 0.01). For the part two proteins (the repair subset), the only significant pathways were “MAPK signalling” (*P*-value = 7.250 × 10^−4^), “WNT signalling” (*P*-value = 1.68 × 10^−2^) and “AMPK signalling” (*P*-value = 1.45 × 10^−2^) based on the above rules (Figure 3F), the same as the part one. Fewer pathway-related proteins in part two suggested that the release of these proteins from the surrounding tissue supports the repair of cerebral ischaemic injury. So, the common pathways between part one and part two were used for next analysis.

### 2.7. Selection and Validation of Candidate Proteins by Western Blot Analysis

**Figure 4:**
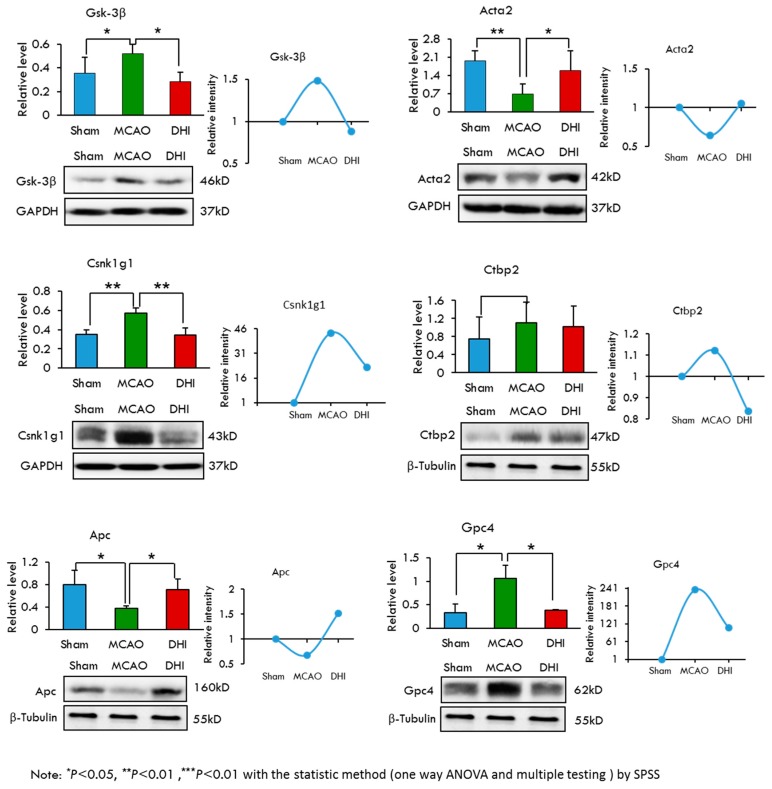
Selection and validation of the six candidates from the WNT signaling pathway for the hippocampus after cerebral ischemic injury and repair by Western blot. The results of Western blotting of the six differentially displayed hippocampus proteome proteins were analyzed with a statistical method (one way ANOVA and multiple testing) by SPSS (Table 3). We show the trends of protein abundance, either from Western blotting or from the label-free analysis.

**Figure 5:**
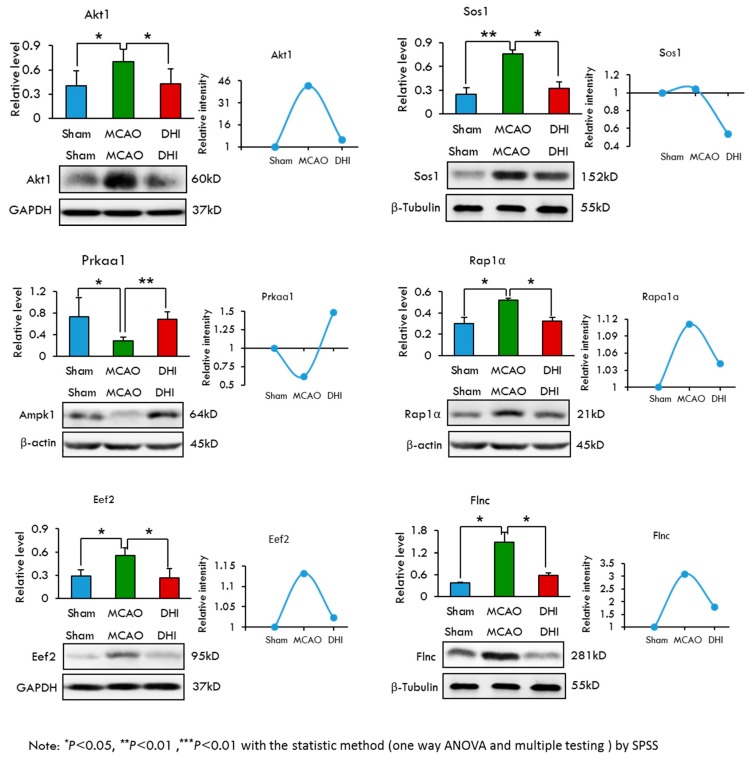
Selection and validation of the six candidates from the AMPK signaling pathway and the MAPK signaling pathway for the hippocampus after cerebral ischemic injury and repair by Western blot. The results of Western blotting of the six differentially displayed hippocampus proteome proteins were analyzed with a statistical method (one way ANOVA and multiple testing) by SPSS (Table 3). We show the trends of protein abundance, either from Western blotting or from the label-free analysis.

To select candidate proteins for validation, we first reviewed the quantitative data and biological information analysis of the proteins to confirm their differential expression at the protein level in the four experiment groups. The selected proteins met our subcellular localization and pathway enrichment criteria as being truly “extracellular region” proteins and belonging to “WNT signalling”, “MAPK signalling” or “AMPK signalling” KEGG pathways. Thus, we narrowed our list down to twelve proteins, including the following: Glypican 4 (Gpc4), Adenomatous polyposis coli protein (Apc), Glycogen synthase kinase-3β (Gsk-3β), Casein kinase I isoform gamma-1 (Csnk1 g1), C-terminal-binding protein 2 (Ctbp2), Anti-α Smooth muscle actin (Acta2), RAC-α serine/threonine-protein kinase (Akt1), Elongation factor 2 (Eef2), 5′-AMP-activated protein kinase catalytic subunit α-1 (Prkaa1), Protein Sos1 (Sos1), Ras-related protein Rap-1 A (Rap1a) and Filamin-C (Flnc). Among these proteins, Glycogen synthase kinase-3β encoded by the Gsk-3β gene was previously reported to be associated with cerebral ischaemic injury [35]. To further validate the regulation of protein levels, we examined the protein abundance via western blotting. Quantity One software was then used to evaluate the Western blotting results by the gray value feature and β-actin, β-tublin and Glyceraldehyde-3-phosphate dehydrogenase (Gapdh) were selected as the loading controls because they are expressed equally in the samples. All of the western blotting WB experiments were repeated five times. The statistical results of Western blotting showed a significant difference (bar charts in Figure 4 and Figure 5).The mean and standard deviation were listed in Table 3. Of the 12 selected proteins, nine exhibited high expression in the MCAO group and low expression in the Sham and MCAO + DHI groups. One protein (Ctbp2) exhibited high expression in the MCAO and MCAO + DHI groups and low expression in the Sham group. The other two proteins displayed high expression in the Sham and MCAO + DHI groups and median expression in the MCAO group (Figure 4 and Figure 5; Table 3). These expression results coincided with the injury and repair capability of the hippocampus after cerebral ischaemic injury. In addition, many studies have suggested that correctly identified protein candidates should participate in relevant interactions. Compared the Western blotting results with the MS label-free expression individually, as expected, the Western blotting results of all 12 proteins indicated gradual expression among the three experimental groups, which was consistent with the label-free results by MS (line graphs in Figure 4 and Figure 5), lending significant confidence to the next validation step.

### 2.8. A Validation Experiment of the Protein Gsk-3β and its Inhibitor

**Figure 6:**
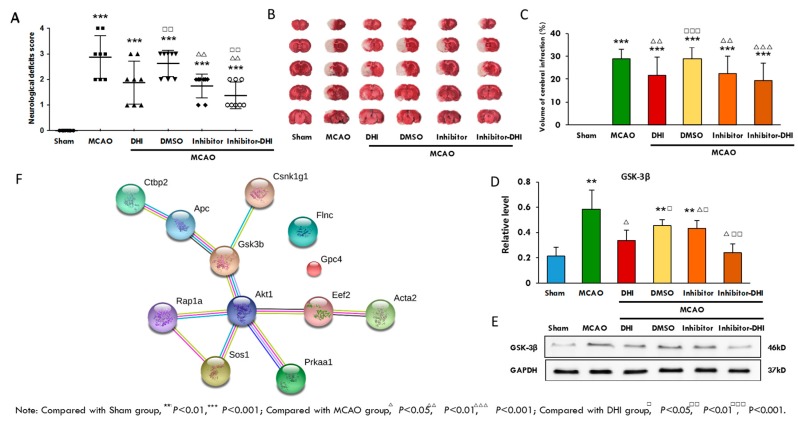
A validation experiment of the candidate protein Gsk-3β and its inhibitor and the network of the 12 candidates. (**A**) Twenty-four hours after MCAO operation, Longa’s Neurological Severity Score from the model group revealed remarkable ischaemic injury, while DHI, Gsk-3β inhibition and DHI + GSK-3β can observably decrease the scores, indicating improved neurological function in MCAO rats (Table 4, a rank sum test was used); (**B**) the brains were stained with TTC; (**C**) the mean infarct volumes in these groups (Table 5, *t*-test was used); (**D**,**E**) validation of the hippocampus proteins from these groups by Western blotting and *t*-test was used to test the quantitative data. The results indicated that the rats treated with the Gsk-3β inhibitor in combination with DHI showed a lower degree of ischaemic injury (**F**) Network of the 12 potential candidate proteins by STRING analysis.

To experimentally validate whether the observed protein expression mediated protection against cerebral ischaemic injury, we evaluated the pharmacological effects of DHI treatment, Gsk-3β inhibition and a combination of both in MCAO rats. As shown in Figure 6A, 24 hours after MCAO operation, Longa’s Neurological Severity Score in the model group revealed remarkable ischaemic injury, while treatment with either DHI or the Gsk-3β inhibitor alone or in combination (DHI + Gsk-3β) resulted in observably lower scores, indicating improved neurological function in MCAO rats. All of the neurological deficits were analysed by a rank sum test (Table 4). Moreover, the rat brains were stained with TTC. The ischaemia produced a marked infarct as a result of the MCAO operation in the serial coronal brain sections. The mean infarct volume in the model group was 28.78 ± 4.25 (%) (*P* < 0.001; Table 5). As shown in Figure 6B and C, DHI treatment, Gsk-3β inhibition and Gsk-3β inhibition combined with DHI treatment significantly reduced the infarct volume (*P* < 0.05) compared to the model group, and Gsk-3β inhibition combined with DHI treatment had the most obvious affect (*P* < 0.01). As shown in Figure 6D,E, Western blot demonstrated that DHI treatment, Gsk-3β inhibition and Gsk-3β inhibition combined with DHI treatment resulted in lower expression of the proteins involved in ischaemic injury compared to the model group.

## 3. Discussion

Our study describes the entire protein expression profile and the changes in the rat hippocampus during the cerebral ischaemic injury and repair processes. The results indicate that 12 proteins can be used as novel biological monitoring indexes of cerebral ischaemic injury and repair. Gsk-3β inhibitors, including lithium ions, attenuate neurodegeneration after trophic withdrawal, oxygen-glucose deprivation, excitotoxicity, and focal ischaemia [36]. Factors that induce Gsk-3β phosphorylation and inhibit the enzyme support neuronal survival [37], whereas an increase in activity of Gsk-3β promotes neuronal degeneration [38]. The results of a Western blot analysis of protein expression and TTC staining of rat brains show that DHI treatment and Gsk-3β inhibition are both able to confer protection against ischaemic injury in a rat MCAO model. Moreover, the effect of the combination of Gsk-3β inhibition with DHI treatment tended to be even more obvious. Therefore, the results suggested that the expression of other important hippocampal proteins was altered due to DHI-mediated protection and cerebral ischaemic injury, revealing other potential therapeutic proteins for cerebral ischaemic stroke.

Since cerebral ischaemic strokes occur when blood flow to the brain stops and the results lead to irreversible damage in some regions [10,11], substantial amounts of proteins from blood flow might leak into the brain after ischaemic injury. Matching our hippocampus dataset to the core human plasma database (PPD) with gene symbol, revealed that there were 60% hippocampus blood-derived proteins (2595 GPs) identified by our studies. Bioinformatics analysis revealed that cell–cell adhesion, cell–cell adherens junction, cadherin binding involved in cell–cell adhesion annotation were significantly enriched (Bonferroni = 7.90 × 10^−58^, 8.87 × 10^−51^, 1.07 × 10^−41^ and FDR = 1.26 × 10^−57^, 7.83 × 10^−51^, 3.58 × 10^−42^) and the annotation cluster enrichment score was up to 52.73, which indicated that these proteins mainly participated in the cell adhesion. Cellular adhesion is essential in maintaining multicellular structure and can link cells in different ways and be involved in signal transduction. Meanwhile, a comparative analysis revealed that nearly 90% of different expression proteins were blood-derived proteins. Subcellular location showed that these proteins in part one were mostly enriched in the extracellular space (Bonferroni = 5.17 × 10^−6^, FDR = 3.47 × 10^−5^), secondly in the cytosol (Bonferroni = 1.78 × 10^−5^, FDR = 1.20 × 10^−4^); the proteins in part two showed no significant location. The results may suggest that DHI is able to repair cerebral ischaemic injury. Function annotation cluster showed that the former contained a great deal of phosphoproteins (Bonferroni = 1.46 × 10^−5^, FDR = 9.45 × 10^−5^) and acetylation proteins (Bonferroni = 6.68 × 10^−5^, FDR = 4.33 × 10^−4^) and the latter only contained significant phosphoproteins (Bonferroni = 0.0079, FDR = 0.058), which suggested that phosphorylation as a core function throughout the whole process of cerebral ischaemic injury both with and without DHI administration.

To understand the interactions among the proteins involved in ischaemic injury and repair in our experimental groups, we investigated the functional network of the 12 selected candidate proteins using STRING analysis (Figure 6F) (Table S1, sheet 8: STRING for candidates). All of the proteins except Gpc4 and Flnc were identified as potential crosstalk components, and Gsk-3β and Akt1 as the specific nodes play an important role in the whole pathological process. Our study indicates that correlation and crosstalk between the different proteins in cerebral ischaemic injury and repair are common. It has been increasingly recognized that under both physiological and pathological conditions, Gsk-3β in the WNT pathway is regulated by the proteins Apc (WNT), Csnk1g1 (WNT), Akt1 (AMPK). Akt1, of the AMPK signalling pathway, is also regulated by the proteins Gsk-3β (WNT), Eef2 (AMPK), Prkaa1 (AMPK), Rap1a (MAPK) and Sos1 (MAPK). Furthermore, the proteins Rap1a and Sos1 of the MAPK pathway interact upon acute damage activation. Although the proteins that cooperate during cerebral ischaemic injury and repair have been studied [7,20], the global network of differential protein expression in both physiological and pathological conditions has not been previously reported.

In fact, the candidate proteins were selected from three significantly enriched signalling pathways: “WNT signalling”, “AMPK signalling” and “MAPK signalling”.

The WNT signalling pathways are a group of signal transduction pathways made up of proteins that pass signals into a cell through cell surface receptors [35,39]. Abnormal WNT signalling causes many types of tumours [40,41] and also controls tissue regeneration in adult bone marrow, skin and intestine [42]. Recent evidence indicates that abnormal WNT signalling might be involved in human brain diseases [43,44,45,46,47]. In the adult brain, different components of WNT signalling are expressed, but little is known about its role in the process. Our studies have indicated that WNT signalling may participate in cerebral ischaemic injury and repair. Furthermore, activation of Gsk-3β, Gpc4, Csnk1g1, Ctbp2, Apc and Acta2 in the WNT signalling pathway has been shown to protect against cerebral ischaemic injury. Activation of the canonical WNT signalling pathway leads to inhibition of Gsk-3β by phosphorylation [48,49,50]. Gsk-3β phosphorylates and thereby regulates the function of metabolic, signalling and structural proteins and transcription factors [51]. In WNT signalling, Gsk-3β forms a multimeric complex with Apc, leading to its degradation mediated by the ubiquitin proteasome system. Gpc4, as a membrane proteoglycan, may play a role in the control of cell division and growth regulation [52]. Csnk1g1 may phosphorylate a large number of proteins in WNT signalling and also regulates fast synaptic transmission mediated by glutamate. Ctbp2 may have the ability to bind to NADH and to a lesser extent NAD+ and typically turn their target genes off [53]. Acta2 is an actin protein, which are highly conserved proteins that are involved in cell motility, structure and integrity [54].

In response to a reduction of intracellular ATP levels, Ampk1 (Prkaa1) activates energy- producing pathways and inhibits energy-consuming processes. Eef2 is an essential factor for protein synthesis. Ampk1 and Eef2 also belong to the AMPK signalling pathway. Sos1, as a membrane-bound protein, may actively function in the transduction of signals that control cell growth and differentiation. Flnc may be involved in reorganizing the actin cytoskeleton in response to signalling events. Rap1a may be involved in the regulation of the expression of the vascular endothelial growth factor receptor KDR at endothelial cell–cell junctions. The latter proteins are part of the MAPK signalling pathway, which is activated when a signalling molecule binds to the receptor on the cell surface and produces some change in the cell, such as cell division. All of the candidate proteins were localized in the extracellular region. In addition, recent evidence indicates that the “AMPK signalling” and “MAPK signalling” pathways might have protective effects in certain brain diseases [55,56,57,58].

In addition, activated Akt1 of the AMPK signalling pathway would have bound the Gsk-3β complex in the presence of WNT overexpression. Akt1 acts as an important regulator of the WNT signalling pathway during the whole process [50]. This finding may indicate a novel concept in crosstalk signalling pathways from directed and connected networks with specific proteins, which were closely related to experimental groups (Figure 6F). Together, our data not only provided 12 important candidates for cerebral ischaemic injury and repair, but also provided insights into the explanation, prevention and treatment of cerebral ischaemia.

## 4. Materials and Methods

### 4.1. Animals

Adult male Sprague Dawley (SD) rats weighing 250–270 g (seven weeks old) were obtained from the Animal Breeding Center of Beijing Vital River Laboratories Company (Beijing, China). All animals were housed individually at 22 ± 2 °C with a relative humidity of 50 ± 10% and a 12 h light/12 h dark cycle. Five rats were kept in each cage. The animals had free access to food and water. The project identification code is 20162002. The experimental procedures were approved by the Academy of Chinese Medical Science's Administrative Panel on Laboratory Animal Care and performed in accordance with institutional guidelines and ethics of committee of China Academy of Chinese Medical Sciences (1 February 2016).

### 4.2. Treatment and Middle Cerebral Artery Occlusion

The permanent middle cerebral artery occlusion (MCAO) model operation by the intraluminal filament method was performed according to a previous method with some modifications [25,26,27]. Briefly, a 4-0 monofilament nylon suture with a round tip was inserted from the left external carotid artery into the lumen of the internal carotid artery to occlude the origin of the MCA. Interruption of blood flow at the occlusion site was continuously monitored by laser-Doppler flowmetry to ensure the adequacy of the MCAO. The whole operation process was completed within 10 min, and the rectal temperature of the rats was maintained at 37 ± 0.5 °C throughout the surgical procedure. The rats were sacrificed 24 h after the MCAO operation. Male Sprague Dawley rats were randomly divided into five groups: a Naive group with 0.9% sodium chloride solution treatment (Naive), a sham group with 0.9% Sodium Chloride Solution treatment (Sham), a middle cerebral artery occlusion (MCAO) group with 0.9% Sodium Chloride Solution treatment (MCAO), a positive control ginaton-treated group (0.45 mL/kg) and a Danhong-treated group (DHI, 0.72 mL/kg). The drugs were delivered by intraperitoneal (i.p.) injection twice, at 15 min and 6 h after the MCAO operation. The Naive rats, which did not undergo the MCAO operation, were injected without any treatment. The Sham rats underwent the procedure described with the exception of insertion of the nylon filament into the MCA. The other three groups underwent the MCAO operation and were injected with the treatments listed above. After neurological defects were determined at 24 h, the rats were studied by analysing the femoral arterial blood and examining the brain infarct volume. In addition to the pharmacodynamic evaluation, the rat hippocampi of the Naive, Sham, MCAO and DHI groups were removed and processed for proteomics analysis.

### 4.3. Gsk-3β Inhibition Validation Experiment

The Gsk-3β Inhibitor XXVI (C₂₁H₁₈N₄O) controls the biological activity of Gsk-3β. This small molecule/inhibitor is primarily used for biochemical applications (Gsk-3β Inhibitor XXVI, Merck KGaA, Darmstadt, Germany, solution in DMSO (50 mg/ML; light yellow solution)). For the drug administration, rats were randomly divided into six groups (*n* = 10 in each group): a Sham group with 0.9% sodium chloride solution treatment (Sham), a middle cerebral artery occlusion (MCAO) group with 0.9% Sodium Chloride Solution treatment (MCAO), an MCAO + DHI-treated group (DHI, 0.72 mL/kg), an MCAO + DMSO group with 0.9% Sodium Chloride Solution treatment (MCAO + DMSO), an MCAO + Gsk-3β inhibition group with 0.9% Sodium Chloride Solution treatment (MCAO + inhibitor) and an MCAO group with both Gsk-3β inhibition and DHI treatment (0.72 mL/kg) (MCAO + inhibitor + DH). Thirty minutes prior to the MCAO experiments, each group was given intracerebral injections. The MCAO + DMSO group was injected intracerebrally with DMSO and the MCAO + inhibitor and MCAO + inhibitor + DHI groups were injected intracerebrally with the Gsk-3β inhibitor. Finally, each group was i.p. injected with the corresponding drug 15 minutes and 6 h after the MCAO operation, as described in the previous section. An appropriate stereotaxic apparatus was used for the intracerebral injections. The skin of the rat skull was removed and the injection location was 3.8 mm posterior to bregma, 2.5 mm right from the midline, and 2.5 mm above the lateral brain ventricle [59]. The injection volume was 2 µL, the injection time was 10 min, and the microsyringe retention time was 20 minutes.

### 4.4. Evaluation of Neurological Deficit

The neurobehavioural dysfunction of rats in all groups was estimated by observers blind to the experiment using Longa’s five-point scale: 0, normal (no neurobehavioural dysfunction); 1, slight (failure to flex left forepaw fully); 2, moderate (circling counterclockwise); 3, severe (leaning to the affected side); and 4, very serious (no autonomous activity and unconsciousness) [32].

### 4.5. Infarct Volume Measurement

The cerebral infarct volumes, as measured by TTC staining, were used to describe the severity of the cerebral ischaemia [28,29,30]. After 24 h of ischaemia, rats were decapitated under anaesthesia, and the brain was removed quickly. Each brain was frozen at −20 °C for 30 min, sliced coronally at 2 mm, stained with 2% TTC (pH 7.4) at 37 °C and fixed in 4% paraformaldehyde fixation solution [60]. After staining with TTC, the normal tissue stained a rose red colour, and the infarct tissue was white. The images of the stained slices were photographed and recorded. The adjusted infarct areas and both the hemisphere areas of each slice were determined by an image analysis system (Image-pro plus 6.0, Media Cybernetics, Rockville, MD, USA). The total infarct volume was calculated as the sum of the infarct areas of the five sections and is presented as a percentage of the volume of the left hemisphere [61].

### 4.6. Rat Hippocampus Sample Preparation

Rat hippocampal proteins were extracted using a Bullet Blender tissue preparation instrument (Next Advance, Averill Park, NY, USA). All procedures were performed at 4 °C. Lysis buffer (8 M urea) was added to the hippocampal tissue at a final ratio of 1:3. After mixing, the tissue was homogenized for 3 min at 4 °C in the Bullet Blender. After centrifugation of the solution (15,000× *g*, 10 min, 4 °C), the protein pellet was discarded and the supernatant was carefully transferred to a new tube for subsequent analysis. The concentration of the extracted proteins was measured by a NanoDrop spectrophotometer (Thermo Fisher Scientific, Waltham, MA, USA) at 280 nm with an extinction coefficient of 1.1 absorbance units [62].

### 4.7. In-Solution Digestion of Hippocampal Proteins

The supernatants were collected from homogenized tissue after centrifugation at 15,000× *g* for 10 min at 4 °C, as described above. The in-solution tryptic digestion was performed using a standard protocol. The lysates were denatured using 8 M urea, reduced with 10 mM dithiothreitol (DTT) and alkylated with 40 mM IAA. Excess iodoacetamide (IAA) was quenched by adding 30 mM of DTT. The urea concentration in the sample solution was reduced to 1 M by diluting the samples with 50 mM NH_4_HCO_3_, and the proteins were digested with trypsin (Promega, Fitchburg, WI, USA) overnight. The protein-to-enzyme ratio was 100:1, and protein digestion was stopped by adding formic acid at a final concentration of 0.1%.

### 4.8. MS Analysis for Peptide Identification

A nanoflow HPLC instrument (EASY-nLC 1000 system, Thermo Fisher Scientific, Waltham, MA, USA) was coupled on-line to a Q-Exactive mass spectrometer with a nanoelectrospray ion source (Thermo Fisher Scientific) for the hippocampus proteome analysis [63]. Chromatography columns were packed in-house with Ultimate XB-C18 3 μm resin (Welch Materials, MD, USA). The peptide mixtures were loaded onto the C_18_-reversed phase column (10 cm length, 75 μm inner diameter) with buffer A (99.5% water and 0.5% FA) and separated with a 75-min linear gradient of 3–100% buffer B (99.5% acetonitrile and 0.5% FA) at a flow rate of 350 nL/min. Including the loading and washing steps, the total time for an LC MS/MS run was approximately 90 min. The electrospray voltage was 2.0 kV. Peptides were analysed by data-dependent MS/MS acquisition with a dynamic exclusion duration of 18 s. In MS1, the resolution was 70,000, the AGC target was 3 × 10^6^, and the maximum injection time was 20 ms. In MS2, the resolution was 17,500, the AGC target was 1 × 10^6^, and the maximum injection time was 60 ms. The scan range was 300–1400 *m*/*z*, and the 20 most intensive precursor ions were selected for MS/MS analysis.

### 4.9. Protein Identification and Label-Free Quantitative Analysis

The raw data were processed using the Proteome Discoverer 1.4 proteomics platform. The fragmentation spectra were searched against the UniProt rat database (20150320) using the Mascot search engine (v 2.2.06) with the precursor and fragment mass tolerances set to 10 ppm and 20 mmu for Q-Exactive (QE) data, respectively. Two missed cleavage sites were allowed, and the minimum peptide length was seven amino acids. The variable modifications included oxidation (M) and acetylation (protein N-terminal), and the fixed modification was carbamidomethyl (C). Additionally, peptide ions were filtered using a cut-off score of 20 and the Percolator algorithm based on *P*-value < 0.01. The false discovery rate (FDR) was set to 1% for peptide identifications.

The label-free quantitative analyses of the hippocampus proteome proteins were estimated by the peak area method, and in-house data filtering and deconvolution were performed as described [64]. Briefly, precursor ions areas were extracted using Proteome Discoverer 1.4 (Thermo Fisher Scientific, Waltham, MA, USA) with 10 ppm mass precision (the experimental *m*/*z* and retention times were recorded for precursor area quantification). Here, we modify the missing values with a drawing random values from a distribution meant to simulate expression below the detection limit by Perseus [65]. Secondly, the areas of each proteins were normalized by number of possible peptides of given proteins. Thirdly, fraction of total proteins areas for each run. The ratios for each protein were normalized by the total identified proteins, and the ratios were used for protein-level quantification. For biological reliability, we performed three biological replicates for each group (for a total of 12 MS runs).

### 4.10. Bioinformatics and Statistical Analysis

An automation tool was developed in-house in Perl and can be run on a Windows Perl environment, facilitating the mapping of a list of protein identities to sequences, running the three prediction software programs mentioned above and creating a report file with consolidated predictions [66]. To evaluate the trend of protein abundance changes, hierarchical clustering was performed and a distance tree was generated using Multi Experiment Viewer (MeV) [67]. To explore the biological functions, subcellular localization, and the related pathways and networks of the identified proteins, Gene Ontology (GO) annotation [68], DAVID Bioinformatics Resources [69], OmicsBean analysis tool [70], STRING analysis software [71] were employed, respectively. 

To visualize the significantly regulated proteins, Statistical Product and Service Solutions (SPSS 19.0) software (IBM Corporation, Armonk, New York, USA) was used. For comparison, *t*-test analysis was performed as appropriate. To evaluate the volume of cerebral infarction, the means ± standard statistical comparisons were made using one-way ANOVA followed by the Newman–Keuls test. A value of *P* < 0.05 was considered to be statistically significant [72]. The evaluations of neurological deficit were analysed using a rank sum test [20].

### 4.11. Western Blotting Analysis

The rat hippocampal proteins were resolved on 10% SDS-PAGE gels and were subsequently transferred onto PVDF membranes (Millipore, Billerica, Massachusetts, USA). After incubation in blocking buffer (0.5% Tween-20 in TBS, 5% BSA) for 1 h at room temperature, the membranes were blotted using antibodies against the target proteins for 1 h at room temperature. Membranes were then washed with TBST (TBS with 0.5% Tween-20) and incubated in 1:1,000 or 1:5,000 diluted 0.5% BSA for 1 h at room temperature (The antibody detail: ab32575, ab32505, ab40778, ab168364, ab8226, 1:1,000, Abcam, Cambridge, MA, USA; sc-133206, 1/5000, Santa Cruz, California, USA; #13256, #2332, #12979, #9832, #2795, #2399, #12409, #15115, #2118, 1:1,000, CST, Danvers, MA, USA). After washing three times with TBST, the bands on the membrane were visualized using an ECL detection system [73].

## 5. Conclusions

In-depth and precise proteomics analysis capable of systematically describing cell and tissue proteomes with high spatiotemporal resolution is both possible and highly valuable [74]. Previous work on cerebral ischaemic injury has demonstrated that there is a lack of systemic research on the relationship between the hippocampus and cerebral ischaemic injury. To explore proteins related to cerebral ischaemic injury, we performed label-free quantitative profiling of the hippocampal proteome in different rat groups by mass spectrometry. By applying tissue extraction, followed by high mass accuracy LC-MS/MS analysis, specific proteins were identified with high confidence. The expression of the proteins in the extracellular system reflected the intracellular regulation of the activation of a synthesis, transport and repair-related pathway, which could be illustrated through GO and KEGG pathway enrichment analysis [69]. Among these biological functions, the most significant class was the “WNT signalling” pathway. The present study provides the first evidence that in vivo activation of “WNT signalling” improves cerebral ischaemic injury and that DHI treatment enhances the long-term potentiation of stimulus-related proteins in the rat hippocampus. Glycogen synthase kinase-3β (Gsk-3β) and RAC-α serine/threonine-protein kinase (Akt1) as the specific nodes are two members of this class that have been previously shown to play an important role during the entirety of the pathological process. Moreover, the activation of other significant proteins and two other signalling pathways are also related to the pathology of cerebral ischaemic diseases. Our study provides evidence of the involvement of many proteins in cerebral ischaemic injury, and analyses of the differentially regulated proteins may result in the discovery of novel candidates for the surveillance of cerebral ischaemic injury.

## Figures and Tables

**Table 1:** The neurological deficit scores of groups from Figure 1A.

Groups	Mean Score
Naive	0.00 ± 0.00
Sham	0.00 ± 0.00
MCAO	2.75 ± 0.43 ***
DHI	1.50 ± 0.50 ***^,^^∆∆^
Ginaton	1.62 ± 0.48 ***^,^^∆∆^

(*** *P* < 0.001 vs. the Sham group; ^∆∆^*P* < 0.01, vs. the MCAO group).

**Table 2:** TTC staining of the brain for the whole experiment.

Groups	Cerebral Infarct Volume (%)
Sham	0.00 ± 0.00
MCAO	29.71 ± 3.47 ***
Ginaton-treated	21.77 ± 7.71 ***^,^^∆^
Danhong-treated	22.25 ± 7.94 ***^,^^∆^

(*** *P* < 0.001 vs. the Sham group; ^∆^*P* < 0.01, vs. the MCAO group).

**Table 3:** The quantity results of Western blot by quantity one software (* *P <* 0.05, ** *P* < 0.01).

Name	Groups	Protein Relative Level(Mean ± SD)
Gsk-3β	Sham	0.352 ± 0.135
	MCAO	0.421 ± 0.163 *
	DHI	0.359 ± 0.169 *
Acta2	Sham	1.693 ± 0.670
	MCAO	0.925 ± 0.527 *
	DHI	1.338 ± 0.762 *
Csk1g1	Sham	0.350 ± 0.048
	MCAO	0.575 ± 0.048 **
	DHI	0.344 ± 0.077 **
Apc	Sham	0.804 ± 0.250
	MCAO	0.371 ± 0.051 *
	DHI	0.743 ± 0.163 *
Gpc4	Sham	0.330 ± 0.188
	MCAO	1.073 ± 0.257 *
	DHI	0.379 ± 0.023 *
Ctbp2	Sham	0.749 ± 0.480
	MCAO	1.103 ± 0.463 **
	DHI	1.018 ± 0.460
Akt1	Sham	0.404 ± 0.187
	MCAO	0.700 ± 0.155 *
	DHI	0.430 ± 0.182 *
Sos1	Sham	0.247 ± 0.083
	MCAO	0.765 ± 0.050 **
	DHI	0.322 ± 0.085 *
Prkaa1	Sham	0.733 ± 0.353
	MCAO	0.282 ± 0.075 *
	DHI	0.684 ± 0.134 **
Eef2	Sham	0.288 ± 0.084
	MCAO	0.558 ± 0.091 *
	DHI	0.265 ± 0.120 *
Flnc	Sham	0.370 ± 0.021
	MCAO	1.477 ± 0.276 *
	DHI	0.587 ± 0.069 *
Rap1a	Sham	0.302 ± 0.058
	MCAO	0.521 ± 0.020 *
	DHI	0.322 ± 0.038 *

**Table 4:** The neurological deficit scores of groups for the Gsk-3β validation experiment.

Groups	Mean Score
Sham	0.00 ± 0.00
MCAO	2.88 ± 0.83 ***
DHI	1.88 ± 0.78 ***
MCAO + DMSO	2.62 ± 0.48 ***^,^^□□^
MCAO + Inhibitor	1.75 ± 0.43 ***^,^^∆∆^
MCAO + Inhibitor + DHI	1.38 ± 0.48 ***^,^^∆∆^^□□^

(*** *P* < 0.001 vs. the Sham group; ^∆∆^ *P* < 0.01 vs. the MCAO group; ^□□^ *P* < 0.01 vs. the DHI group).

**Table 5:** TTC staining of the brain for the Gsk-3β validation experiment.

Groups	Cerebral Infarct Volume (%)
Sham	0.00 ± 0.00
MCAO	28.78 ± 3.92 ***
DHI	21.79 ± 4.54 ***^,^^∆∆^
MCAO + DMSO	28.75 ± 3.28 ***^,^^□□□^
MCAO + inhibitor	22.62 ± 4.26 ***^,^^∆∆^
MCAO + inhibitor + DHI	19.42 ± 3.48 ***^,^^△△△^

(*** *P* < 0.001 vs. the sham group; ^∆∆^ *P* < 0.01, ^△△△^ *P* < 0.001 vs. the MCAO group or MCAO + DMSO group; ^□□□^ *P* < 0.001 versus the DHI group).

## Supplementary Materials

Supplementary materials can be found at www.mdpi.com/1422-0067/18/7/1355/s1.
